# COVID-19 patients with hypertension are at potential risk of worsened organ injury

**DOI:** 10.1038/s41598-021-83295-w

**Published:** 2021-02-12

**Authors:** Fei Xia, Mingwei Zhang, Bo Cui, Wei An, Min Chen, Ping Yang, Tao Qin, Xiaoyang Zhou, Yaling Liao, Xin Xu, Shiguo Liu, Kuangyu Li, Qin Zhou, Keke Wang, Guangxu Hu, Ming Du, Songrui Chen, Yafang Zhang, Wei Wei, Ming Xiang, Jianjun Zhang

**Affiliations:** 1 Department of Clinical Pharmacy, Hubei No. 3 People’s Hospital of Jianghan University (the Third People’s Hospital of Hubei Province), Wuhan, China; 2 Department of Cardiology, Renmin Hospital of Wuhan University, Cardiovascular Research Institute of Wuhan University, Wuhan, China; 3 Department of Pharmacy, The First Affiliated Hospital of China Medical University, Shenyang, Liaoning China; 4 Department of Pharmacology, School of Pharmacy, Tongji Medical College, Huazhong University of Science and Technology, Wuhan, China

**Keywords:** Viral infection, Epidemiology

## Abstract

In less than 6 months, COVID-19 spread rapidly around the world and became a global health concern. Hypertension is the most common chronic disease in COVID-19 patients, but its impact on these patients has not been well described. In this retrospective study, 82 patients diagnosed with COVID-19 were enrolled, and epidemiological, demographic, clinical, laboratory, radiological and therapy-related data were analyzed and compared between COVID-19 patients with (29 cases) or without (53 cases) hypertension. The median age of the included patients was 60.5 years, and the cohort included 49 women (59.8%) and 33 (40.2%) men. Hypertension (31 [28.2%]) was the most common chronic illness, followed by diabetes (16 [19.5%]) and cardiovascular disease (15 [18.3%]). The most common symptoms were fatigue (55 [67.1%]), dry cough (46 [56.1%]) and fever ≥ 37.3 °C (46 [56.1%]). The median time from illness onset to positive RT-PCR test was 13.0 days (range 3–25 days). There were 6 deaths (20.7%) in the hypertension group and 5 deaths (9.4%) in the nonhypertension group, and more hypertensive patients with COVID-19 (8 [27.6%]) than nonhypertensive patients (2 [3.8%]) (*P* = 0.002) had at least one comorbid disease. Compared with nonhypertensive patients, hypertensive patients exhibited higher neutrophil counts, serum amyloid A, C-reactive protein, and NT-proBNP and lower lymphocyte counts and eGFR. Dynamic observations indicated more severe disease and poorer outcomes after hospital admission in the hypertension group. COVID-19 patients with hypertension have increased risks of severe inflammatory reactions, serious internal organ injury, and disease progression and deterioration.

## Introduction

In 2020, COVID-19 spread rapidly around the globe, and the disease remains an international public health concern^1^. As a betacoronavirus in the 2β lineage, SARS-CoV-2 shares 79.5% sequence identity with SARS-CoV and 96% identity to a bat coronavirus at the whole-genome level^2^. By September 2020, the virus had spread to nearly 200 countries and regions, infecting more than 28 million people and killing nearly 1 million.

SARS-CoV-2 can infect multiple systems and organs through the binding of its spike protein to angiotensin converting enzyme II (ACE2); the SARS-CoV-2 spike has a higher affinity than the SARS-CoV spike for ACE2. As a functional receptor, the ACE2 protein is abundantly expressed in the epithelia of the human lung and small intestine^3^. Thus, the clinical spectrum of SARS-CoV-2 infection is broad, including fever, cough, dyspnea, decreased leukocyte counts or white blood cell counts, mild upper respiratory tract illness, and severe viral pneumonia^4^. System or organ malfunction, including shock, acute respiratory distress syndrome (ARDS), acute cardiac injury, and even death, can also occur in severe cases^5^. It is worth nothing that variations in the ACE system, including in ACE1 and ACE2, also contribute to the occurrence of hypertension^6^. However, the specific effects of SARS-CoV-2 infection on individuals with hypertension are unknown, and it is unclear whether hypertensive individuals with COVID-19 are at greater risk of serious outcomes^4,7,8^. In this study, we aimed to describe the epidemiology, clinical features, and pharmacotherapy response of COVID-19 inpatients and to further compare the available data between hypertensive and nonhypertensive patients admitted to Hubei No. 3 People’s Hospital of Jianghan University (the Third People’s Hospital of Hubei Province).

## Methods

### Study design and participants

This retrospective, single-center, observational study was conducted at Hubei No. 3 People’s Hospital of Jianghan University, Wuhan, China. Patients were hospitalized from Dec 31, 2019, to Feb 01, 2020, and the final date of follow-up was February 08, 2020. Hubei No. 3 People’s Hospital of Jianghan University is a designated COVID-19 hospital with more than 1500 beds. The diagnosis of COVID-19 was made based on the current New Coronavirus Pneumonia Prevention and Control Program (6th edition, in Chinese) released by the National Health Commission of China^9^ and was indicated by suspected symptoms, chest CT results and SARS-CoV-2 positivity on quantitative RT-PCR. Throat-swab specimens from all patients were collected at admission and kept in virus transport medium. Briefly, SARS-CoV-2 detection in respiratory specimens was conducted by the Chinese Center for Disease Control and Prevention, the Chinese Academy of Medical Science, the Academy of Military Medical Sciences, and the Wuhan Institute of Virology, the Chinese Academy of Sciences using real-time RT-PCR or next-generation sequencing technology. Other respiratory viruses, including influenza A virus (H1N1, H3N2, H7N9), influenza B virus, respiratory syncytial virus, parainfluenza virus, adenovirus, SARS coronavirus (SARS-CoV), and MERS coronavirus (MERS-CoV), were also detected by real-time RT-PCR. Sputum or endotracheal aspirates were also examined to identify potential pathogenic bacteria or fungi. Chest computed tomographic (CT) scans were carried out at least twice for each patient.

Patients hospitalized with COVID-19 are clinically classified as having mild, moderate, or severe disease, defined as follows: mild cases: mild clinical symptoms, no manifestations of pneumonia on imaging; moderate cases: symptoms such as fever and respiratory tract symptoms and imaging showing signs of pneumonia; severe cases: respiratory distress, respiratory rate ≥ 30 breaths/min, SpO_2_ ≤ 93% at rest, or PaO_2_/FIO_2_ ratio ≤ 300. In addition, cases with > 50% lesions progressing within 24 to 48 h on pulmonary imaging are considered severe.

### Ethical approval

The research protocol was reviewed and approved by the Ethics Committee of Hubei No. 3 People’s Hospital of Jianghan University (202004). All procedures were carried out in accordance with the ethical standards of the institutional and/or national research committee and the 1964 Declaration of Helsinki. All patients had completed treatment at the beginning of the study, and the study did not interfere with diagnosis or treatment in any case. Therefore, the need for informed consent was waived by the Ethics Committee of Hubei No. 3 People’s Hospital of Jianghan University. In addition, all patient privacy and data were respected and protected.

### Data collection

Eighty-two patients hospitalized with COVID-19 were included in this retrospective study, and 29 of these patients also had hypertension. Epidemiological, demographic, clinical, laboratory, X-ray and chest CT scan, treatment, and outcome data were extracted from electronic medical records with data collection forms. Eight researchers, including physicians and clinical pharmacists, reviewed the data collection forms and examined the data independently three times. COVID-19 patients were divided into two groups according to the presence or absence of hypertension. Hypertension was defined as clinic systolic blood pressure ≥ 140 mmHg and/or diastolic blood pressure ≥ 90 mmHg without the use of antihypertensive medications. Subjects with a blood pressure < 140/90 mmHg but with a history of hypertension and who were taking antihypertensive medication at the time of admission were also included in the hypertension group^10^.

### Statistical analysis

Categorical variables are reported as frequencies and percentages. Continuous variables are reported as mean (SD), median, and interquartile range (IQR) values. Comparisons of quantitative variables between groups were performed by the Wilcoxon rank sum test. Categorical variables are expressed as numbers (%) and were compared between groups using χ^2^ or Fisher’s exact tests. A two-sided p-value less than 0.05 was considered statistically significant. Statistical analysis was performed in SPSS (version 21.0).

## Results

### Baseline characteristics of hospitalized COVID-19 patients on admission

**Figure 1:**
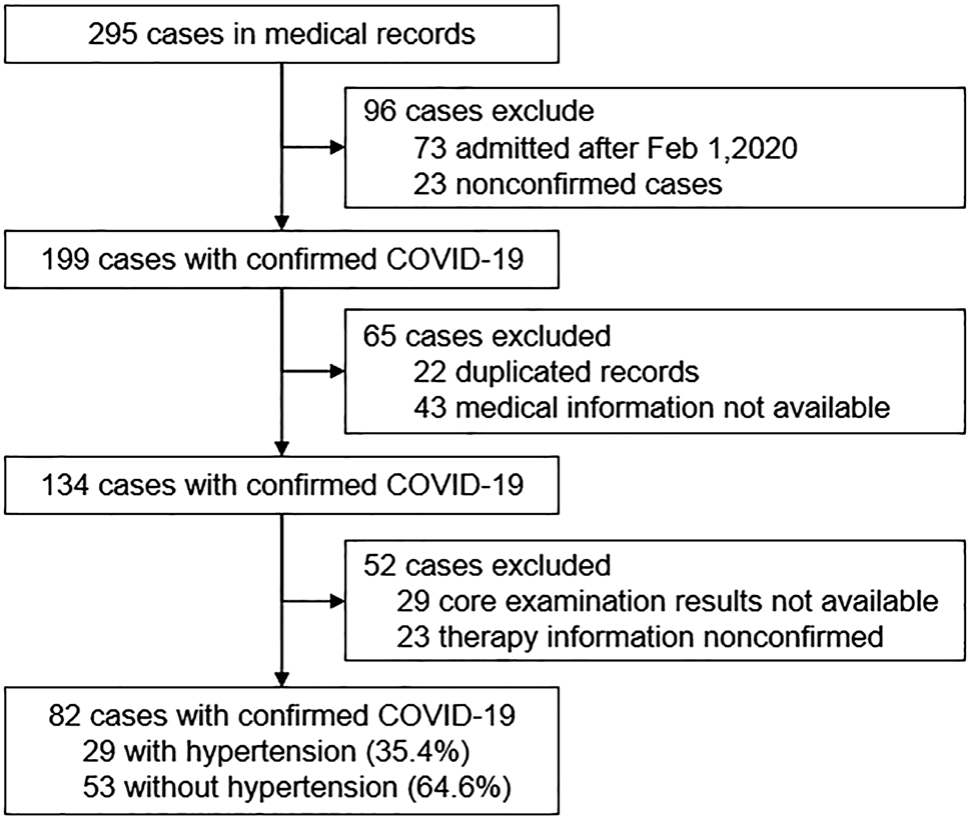
Flow chart for participant inclusion.

**Figure 2:**
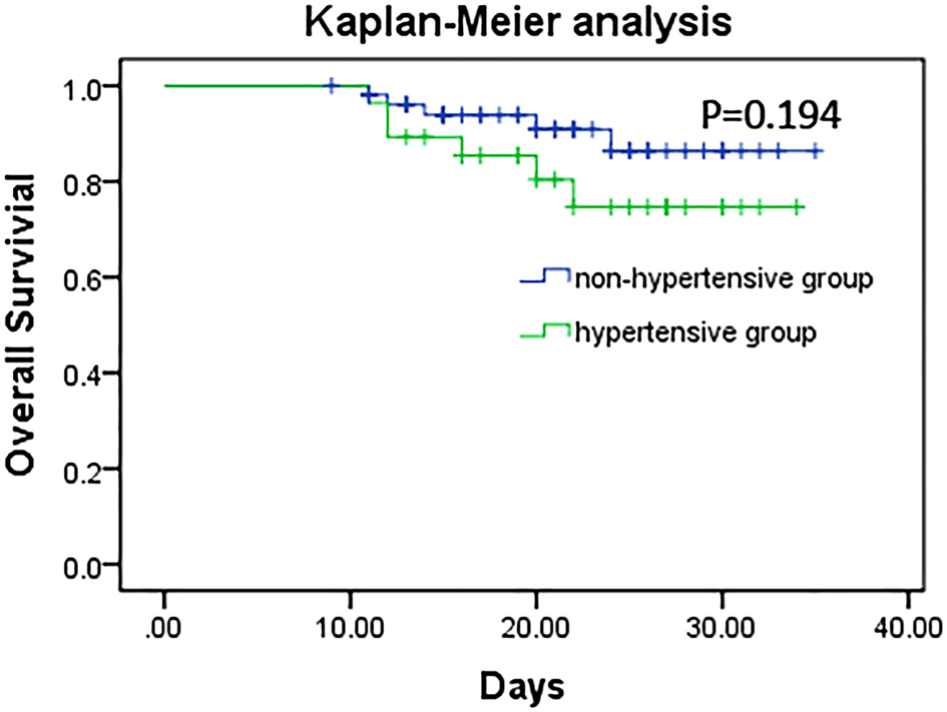
Survival analysis between the hypertension group and the nonhypertension group.

Figure 1 shows the flow chart for participant inclusion. Briefly, 295 cases with dates from Dec 31, 2019, to Feb 28, 2020, in the medical record system were initially screened, of which 213 were considered ineligible, including 73 cases admitted after 01 Feb, 23 cases without confirmed COVID-19 diagnosis, 22 duplicate records, 43 cases without available medical information, and 52 cases with missing core examination or therapy information. Ultimately, 82 patients were included in this study. The median age of the included patients was 60.5 years, and 49 (59.8%) were women (Table 1). The median time from first symptom to hospital admission was 7.0 days. Hypertension (29 [35.4%]) was the most common coexisting chronic illness, followed by diabetes (16 [19.5%]) and cardiovascular disease (15 [18.3%]). Fatigue (55 [67.1%]), dry cough (46 [56.1%]), and fever ≥ 37.3 °C (46 [56.1%]) were the most common symptoms. The median time from illness onset to positive RT-PCR test was 13.0 days (range 3–25 days). A significant difference in clinical types on admission was observed (*P* < 0.001) between the two groups. There was no significant difference in mortality rate between the hypertension group (6 [20.7%]) and the nonhypertension group (5 [9.4%]). Furthermore, no significant difference between the two groups was observed in the survival analysis (Fig. 2).

**Table 1:** Demographics and clinical characteristics of patients with COVID-19 on admission.

	All patients (n = 82)	Hypertension group (n = 29)	Nonhypertension group (n = 53)	*χ*^2^*/Z*	*P*
**Age, years**	60.5 (46.8–69.0)	66.0 (56.5–69.0)	57.0 (40–68.5)	− 10.61	< 0.001
Female age	60.0 (44.0–69.0)	66.5 (57.8–71.5)	63.5 (56.8–70.3)	− 14.798	< 0.001
male age	61.0 (46.5–69)	63.0 (53.0–69.0)	57.5 (44.0–71.5)	− 0.967	0.334
**Sex**					
Female	49 (59.8)	14 (48.3)	19 (35.8)	1.204	0.273
Male	33 (40.2)	15 (51.7)	34 (64.2)		
**Clinical type on admission**					
Mild	3 (3.7)	0 (0.0)	3 (5.7)	43.931	< 0.001
Moderate	68 (82.9)	23 (79.3)	45 (84.9)		
Severe	11 (13.4)	6 (20.1)	5 (9.4)		
**Illness onset to hospital admission, days**	7.0 (4.0–10.0)	7 (5.5–10)	7 (4–10)	− 1.129	0.259
**Chronic illnesses**					
Hypertension	29 (35.4)	29 (100.0)	0 (0)	–	–
Cardiovascular disease	15 (18.3)	9 (31.0)	6 (11.3)	4.814	0.028
Diabetes	16 (19.5)	8 (27.6)	8 (15.1)	1.862	0.172
COPD	7 (8.5)	4 (13.8)	3 **(**5.7**)**	1.568	0.210
Malignancy	1 (1.2)	0 (0.0)	1 (1.9)	0.547	0.459
Digestive system disease	3 (3.7)	2 (6.9)	1 (1.9)	1.318	0.251
Cerebrovascular disease	2 (2.4)	2 (6.9)	0 (0.0)	3.747	0.054
Nervous system disease	3 (3.7)	1 (3.4)	2 (3.8)	0.006	0.941
Chronic liver disease	3 (3.7)	3 (10.3)	0 (0.0)	5.622	0.018
More than one disease	10 (12.2)	8 (27.6)	2 (3.8)	9.926	0.002
**Signs and symptoms**					
Fever (≥ 37.3 °C)	46 (56.1)	13 (44.8)	33 (62.2)	2.314	0.128
≥ 38 °C	28 (34.1)	6 (20.7)	22 (41.5)	3.361	0.057
Fatigue	55 (67.1)	16 (55.2)	39 (73.6)	2.877	0.090
Dry cough	46 (56.1)	13 (44.8)	33 (62.3)	2.314	0.128
Shortness of breath	23 (28.0)	7 (24.1)	16 (30.2)	0.34	0.560
Diarrhea	15 (18.3)	7 (24.1)	8 (15.1)	1.026	0.311
Anorexia	10 (12.2)	1 (3.4)	9 (17.0)	3.167	0.075
Myalgia	9 (15.1)	3 (10.3)	6 (11.3)	0.018	0.893
Expectoration	20 (24.3)	10 (34.4)	10 (18.9)	2.478	0.115
Pharyngalgia	4 (4.9)	2 (6.9)	2 (3.8)	0.389	0.533
Nausea or vomiting	7 (8.5)	2 (6.9)	5 (9.4)	0.155	0.694
Dyspnea	5 (6.1)	2 (6.9)	3 (5.7)	0.049	0.824
More than three signs and symptoms	40 (48.9)	17 (58.6)	23 (434)	1.739	0.187
**Time from illness onset to positive RT-PCR test, days**	13 (6.0–19.0)	13.0 (6.0–20.0)	12.5 (6.0–15.5)	− 0.372	0.743
**Deaths**	11 (13.4)	6 (20.7)	5 (9.4)	2.044	0.153
Female	5 (4.5)	3 (10.3)	2 (3.8)	0.100	0.752
Male	6 (5.4)	3 (10.3)	3 (5.7)		
Time from onset to hospital admission, days	7.0 (5.0–10.0)	7.0 (5.0–8.0)	10 (5.5–20)	− 0.746	0.456
Time from admission to death, days	6.0 (4.0–12.0)	7.5 (3.0–12.5)	4.0 (2.5–9.5)	− 0.737	0.461
Time from onset to death, days	13.0 (10.5–19.5)	13.5 (10.5–19.5)	11.0 (10.5–28.5)	− 0.184	0.854

Data are presented as the median (IQR) or n/N (%), where N is the total number of patients with available data. *p* values comparing patients with or without hypertension cases are from *χ*^2^, Fisher’s exact test, or Wilcoxon rank sum test.

*COVID-19* coronavirus disease 2019, *IQR* interquartile range, *COPD* Chronic Obstructive Pulmonary Disease.

### Laboratory markers in patients with or without hypertension

Major laboratory markers were recorded at hospital admission for all patients (Table 2). There were no significant differences in routine blood analysis parameters between the hypertension and nonhypertension groups. However, the hypertension group exhibited higher white blood cell counts (4.9 × 10^9^/L), neutrophil counts (3.9 × 10^9^/L), and neutrophil percentages (79.3%) and lower lymphocyte counts (0.72 × 10^9^/L) and lymphocyte percentages (12.5%). Moreover, the median SAA in the hypertension group reached 630.1 mg/L, which was approximately double that (373.5 mg/L) in the nonhypertension group. Similarly, the median CRP level (79.2 mg/L) also exceeded that of the nonhypertension group (57.8 mg/L). In blood chemistry assays, no significant changes were found in AST (32.4 U/L vs 34.1 U/L), ALT (23.0 U/L vs 26.3 U/L), and GGT (29.0 U/L vs 30.3 U/L) between the two groups, and all medians were within the normal ranges. For renal injury, eGFR in hypertension group with COVID-19 was significantly decreased compared with that of nonhypertensive group (77.0 mL/min/1.73 m^2^ vs 113.0 mL/min/1.73 m^2^) (*P* = 0.017), while higher levels of urea nitrogen (4.7 mmol/L vs 4.2 mmol/L) and serum creatinine (70.5 μmol/L vs 57.0 μmol/L) were observed in the hypertension group. There were also elevated levels of NT-proBNP (166 ng/L vs 26 ng/L), lactate dehydrogenase (263.0 U/L vs 240.0 mU/L), and creatine kinase (143.0 U/L vs 64.0 U/L), with a significant increase in NT-proBNP in the hypertension group with COVID-19.

**Table 2:** Laboratory findings of patients infected with COVID-19 on admission.

	Normal range	All patients (n = 82)	Hypertension group (n = 29)	Nonhypertension group (n = 53)	*Z*	*P*
**Blood routine tests**						
WBC, × 10^9^/L	3.5–9.5	4.7 (3.5–6.7)	4.9 (3.7–7.4)	4.5 (3.3–6.6)	− 1.093	0.274
Neutrophil counts, × 10^9^/L	1.8–6.3	3.2 (2.2–5.2)	3.9 (2.7–6.3)	3.1 (2.2–4.9)	− 1.544	0.122
N (%),	40–75	76.0 (63.8–84.3)	79.3 (69.2–85.7)	71.6 (62.9–83.4)	− 1.852	0.064
Lymphocyte counts, × 10^9^/L	1.1–3.2	0.8 (0.6–1.1)	0.72 (0.59–0.10)	0.81 (0.56–1.26)	− 0.652	0.515
L (%)	20–50	14.1 (9.1–19.2)	12.5 (8.6–17.3)	16.8 (12.2–21.3)	− 1.625	0.098
Platelets, × 10^9^/L	125–350	192.0 (127.0–225.0)	195 (165–293)	188 (130–225)	− 0.492	0.623
Hemoglobin, g/L	130·0–175	127.0 (119.0–135.0)	129.5 (114.8–137)	126.5 (120–134.8)	− 0.067	0.947
**Infection-related biomarkers**						
PCT, ng/mL	0.04–0.25	0.1 (0.0–0.2)	0.07 (0.04–0.18)	0.05(0.04–0.1)	− 1.679	0.093
SAA, mg/L	0.1–10	501.0 (111.3–102.0)	630.1 (185.3–1010.5)	373.5 (48.8–777.5)	− 1.509	0.131
CRP, mg/L	0–5	67.5 (27.2–102.0)	79.2 (33.5–129.1)	57.8 (20.4–83.1)	− 1.802	0.072
ESR, mm/h	0–20	47.8 (23.3–80.5)	50.5 (24.9–88.0)	47.8 (19–70)	− 0.723	0.470
**Blood biochemistry**						
AST, U/L	8–40	33.6 (26.2–51.2)	32.4 (24.2–49.8)	34.1 (26.8–57.0)	− 0.982	0.326
ALT, U/L	5–35	26.1(17.0–40.0)	23.0 (18.1–42.0)	26.3 (14.7–39.4)	− 0.630	0.529
GGT, U/L	0–50	30.2 (18.0–50.6)	29.0 (22.6–48.3)	30.3 (16.2–55.4)	− 0.583	0.560
Serum urea nitrogen, mmol/L	3.5–7.2	4.4 (3.2–4.5)	4.7 (3.4–5.9)	4.2 (3.2–4.9)	− 1.324	0.185
Serum creatinine, μmol/L	44–120	60.0 (49.0–79.3)	70.5 (51.8–80.8)	57.0 (49.0–79)	− 1.473	0.142
eGFR, mL/min/1.73 m^2^	> 90	102.0 (84.0–119.3)	77.0 (35.3–100.7)	113.0 (91.9–125.9)	− 2.387	0.017
Lactate dehydrogenase, U/L	120–250	249.5 (200.8–353.8)	263.0 (235–384.5)	240.0 (193.0–318.5)	− 1.042	0.297
Creatine kinase, U/L	50–310	97.5 (55.7–179.8)	143.0 (84.0–220.0)	64.0 (50.0–154.0)	− 1.732	0.083
NT-proBNP, ng/L	25–500	115 (26–372)	166 (70–1293)	26 (20–120.5)	− 10.252	< 0.001
K, mmol/L	3.5–5.5	3.7 (3.4–4.0)	3.6 (3.5–4.1)	3.7 (3.5–3.9)	− 0.635	0.525
Ca, mmol/L	2.0–2.6	2.1 (2.0–2.2)	2.1 (2.0–2.1)	2.1 (2.0–2.2)	− 0.112	0.911
Albumin, g/L;	40–55	39.0 (35.8–43.3)	36.4 (34.6–39.9)	40.0 (36.6–44.6)	− 2.218	0.026
Glucose, mmol/L	3.9–6.1	7.54 (6.26–9.23)	7.8 (7.4–9.4)	6.5 (5.5–10.0)	− 1.583	0.113
Total bilirubin, μmol/L	3.4–20.5	9.9 (8.4–13.5)	9.8 (8.6–12.2)	10.0 (8.0–13.8)	− 0.159	0.874
Direct bilirubin, μmol/L	0–6	3.7 (3.2–4.7)	3.5 (3.2–4.7)	3.9 (3.1–4.8)	− 0.817	0.413
Total bile acid, μmol/L	0–12	4.4 (2.5–6.9)	3.8 (2.5–6.3)	5.3 (2.5–9.1)	− 1.141	0.254
**Coagulation function**						
D-dimer, μg/mL	0·0–1.5	0.5 (0.3–1.1)	0.5 (0.3–1.0)	0.5 (0.3–1.4)	− 0.157	0.875
Prothrombin time, S	9–14	10.4 (7.9–11.1)	10.6 (9.7–11.1)	10.8 (10.3–11.5)	− 1.109	0.267
Activated partial thromboplastin time, S	20–40	27.4 (11.7–31.9)	29.7 (24.7–39.2)	28.2 (26.9–32.0)	− 0.802	0.422
Fibrin(-ogen) degradation products, mg/L	0–5	3.7 (2.5–5.7)	3.7 (3.0–5.5)	3.7 (1.7–8.7)	− 0.217	0.828

Data are expressed as the median (IQR) or n/N (%), where N is the total number of patients with available data. *p* values comparing patients with or without hypertension are from *χ*^2^, Fisher’s exact, or Wilcoxon rank sum tests.

*COVID-19* coronavirus disease 2019, *IQR* interquartile range, *WBC* white blood cell, *PCT* procalcitonin, *SAA* serum amyloid A, *CRP* C-reactive protein, *ESR* erythrocyte sedimentation rate, *AST* aspartate aminotransferase, *ALT* alanine aminotransferase, *GGT* glutamine transpeptidase, *eGFR* estimated glomerular filtration rate, *NT-proBNP* N-terminal pro-brain natriuretic peptide.

### Imaging findings

Of all 82 nonmedical COVID-19 patients on admission (Table 3), 14 (17.1%) patients showed unilateral pneumonia, and 64 (78.3%) developed bilateral pneumonia. Twenty-eight (34.2%) patients showed patchy shadows, while 22 (26.8%) patients showed multiple patchy shadows. Twenty-two (26.8%) patients also displayed ground glass opacities. In addition, hydrothorax occurred in 9 (11.0%) patients.

**Table 3:** Chest X-ray and CT findings of COVID-19 patients on admission.

	All patients (n = 82)	Hypertension group (n = 29)	Nonhypertension group (n = 53)	*χ*^2^	*P*
Bilateral pneumonia	64 (78.3)	23 (79.3)	41 (77.4)	0.042	0.838
Unilateral pneumonia	14 (17.1)	4 (13.8)	10 (18.9)	0.341	0.559
Patchy shadows	28 (34.2)	9 (31.0)	19 (35.8)	0.249	0.618
Multiple patchy shadows	22 (26.8)	11 (37.9)	11 (20.7)	2.871	0.093
Ground glass opacity	4 (4.9)	1 (3.4)	3 (5.7)	0.198	0.657
Hydrothorax	9 (11.0)	3 (10.3)	6 (11.3)	0.018	0.892

Data are expressed as the median (IQR) or n/N (%), where N is the total number of patients with available data. *p* values comparing patients with or without hypertension are from *χ*^2^ or Fisher’s exact tests.

*COVID-19* Coronavirus Disease 2019, *CT* computed tomography.

### Organ injuries and main treatments

On admission, common complications among 82 patients included ARDS (9 [11.0%]), sepsis (3 [3.7%]), acute renal injury (1 [1.2%]), and acute respiratory injury (1 [1.2%]) (Table 4). Although the differences between the two groups failed to reach statistical significance on hospital admission, the laboratory outcomes suggested that patients with hypertension had higher risks of multiple organ injuries in the kidney, heart, and lung. For COVID-19 treatment, 63 (76.8%) patients received antiviral therapy, including oseltamivir (56 [50.9%]), arbidol (46 [41.8%]), lopinavir/ritonavir (42 [38.2%]), and ganciclovir (28 [25.4%]). Many patients received glucocorticoid therapy (73 [89.0%]) and antibacterial therapy (55 [67.1]), including carbapenems (22 [20.0%]), quinolones (33 [30.0%]), and cephalosporins (14 [12.7%]). Fifty-five (67.1%) patients received both antiviral and antibacterial therapies. Oxygen therapy (24 [29.3%]) and immune globulin γ treatment (42 [51.3%]) were also applied.

**Table 4:** Complications and treatments of COVID-19 patients.

	All patients (n = 82)	Hypertension group (n = 29)	Nonhypertension group (n = 53)	*χ*^2^	*P*
**Complications**					
ARDS	9 (11.0)	5 (17.2)	4 (7.5)	1.803	0.179
Sepsis	3 (3.7)	2 (6.9)	1 (1.9)	1.402	0.236
Acute renal injury	1 (1.2)	1 (3.4)	0	1.893	0.169
Acute respiratory injury	1 (1.2)	1 (3.4)	0	1.893	0.169
**Treatment**					
Antiviral therapy	63 (76.8)	22 (75.9)	41 (77.4)	0.024	0.878
Two antiviral chemicals	28 (34.1)	11 (37.9)	17 (32.1)	0.286	0.593
Three antiviral chemicals	16 (19.5)	6 (20.7)	10 (18.9)	0.040	0.842
Glucocorticoid therapy	73 (89.0)	26 (89.7)	47 (88.7)	0.018	0.892
Antibacterial therapy	55 (67.1)	18 (62.7)	37 (69.8)	0.509	0.476
Anti-virus and antibacterial treatment	34 (41.5)	14 (48.3)	20 (37.7)	0.858	0.354
Oxygen therapy	24 (29.3)	11 37.9)	13 (24.5)	1.626	0.202
Immune globulin γ treatment	42 (51.3)	15 (51.7)	27 (50.9)	0.005	0.946
Antihypertensive drugs	–	25 (86.2)	–	–	–

Data are expressed as n/N (%), where N is the total number of patients with available data. *p* values comparing patients with or without hypertension cases are from *χ*^2^ or Fisher’s exact test.

*COVID-19* Coronavirus Disease 2019, *ARDS* Acute respiratory distress syndrome.

### Dynamic characteristics of laboratory parameters in COVID-19 patients with or without hypertension

**Figure 3:**
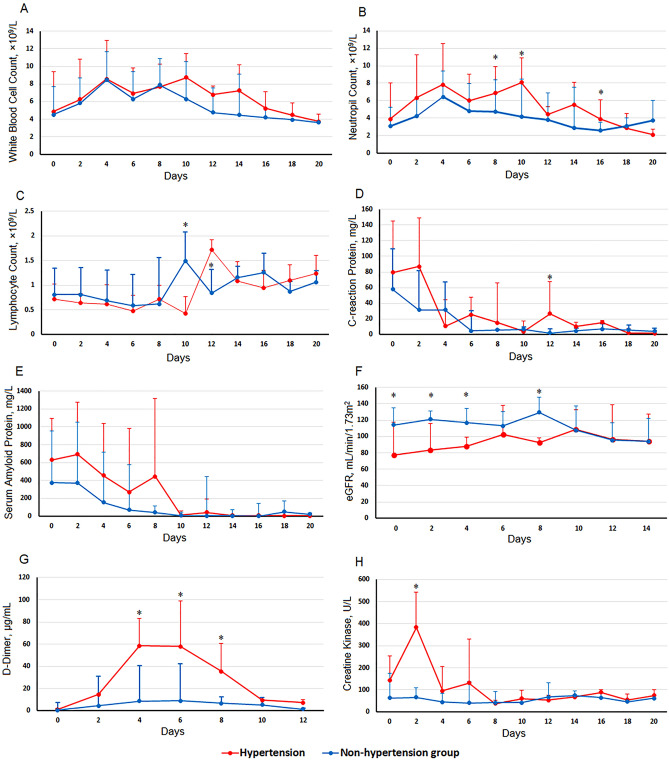
Timelines of laboratory markers from hospital admission for COVID-19. Figure shows dynamic changes in white blood cell counts (**A**), neutrophil counts (**B**), lymphocyte counts (**C**), C-creation protein (**D**), serum amyloid A (**E**), eGFR (**F**), D-dimer (**G**), and creatine kinase (**H**). For eGFR and D-dimer, continuous data at 2-day intervals were available for 14 days and 12 days, respectively. *P < 0.05 for hypertension group vs nonhypertension group.

The dynamic laboratory features of COVID-19 patients, including 8 clinical parameters related to hematology, infection, coagulation function, and internal organ injury, were traced from hospital admission to 20 days in the hospital at a 2-day interval on the basis of no significant difference observed at hospital admission between the two groups (Table 1). As shown in Fig. 3A and B, white blood cell and neutrophil counts were higher in the hypertension group than in the nonhypertension group for almost the whole duration of hospitalization. Most patients had notable lymphopenia, with a 2-day delay in lymphocyte count increasing to the normal range compared to the nonhypertension group (Fig. 3C). Both CRP and SAA were higher in the hypertension group until 10 days after admission (Fig. 3D and E). The eGFR of patients with hypertension was markedly lower than that of nonhypertensive patients, maintaining an overall slow increase from the day of admission but a slight decrease from its peak on day 10; by contrast, there was a reduction of approximately 30 mL/min/1.73 m^2^ in the eGFR of the nonhypertension group from days 8 to 14 (Fig. 3F). The creatine kinase level in the hypertension group was higher on admission, increased to approximately 400 U/L on day 2, and then decreased (Fig. 3H). Similarly, the level of D-dimer in the hypertension group continued to increase until day 4 after admission or day 11 from onset and then declined after day 6 (Fig. 3G).

## Discussion

In this investigation, we studied the epidemiology, clinical characteristics, and treatment of COVID-19, with a particular focus on potential differences in the disease course in the hypertensive population. Among the 82 included COVID-19 patients, there were 11 deaths (13.4%), which was consistent with the overall COVID-19 mortality rate at Jinyintan Hospital during the same period^8^. Nevertheless, national data showed that the case fatality had dropped to 2.3% by February 11^11^, suggesting that SARS-CoV-2 was more lethal at the early stage of the outbreak in January^12^.

Previously, hypertension has been identified as the most common chronic illness in more COVID-19 patients^4,5,13,14^. ACE2 is the receptor that mediates SARS-CoV-2 invasion in COVID-19, identical to SARS-CoV transmission, via the spike (S) glycoprotein–ACE2 binding pathway^15–18^. After infection, the ACE2 level was found to be reduced due to binding with the spike protein of SARS-CoV^19^, suggesting that SARS-CoV-2 may also reduce the level of ACE2 in infected cells, resulting in an imbalance between ACE1 and ACE2. Second, the renin-angiotensin II-aldosterone axis has been traditionally recognized as a key regulator of blood pressure in the development of hypertension, with AngII levels regulated by ACE. The balance between ACE1 and ACE2 is crucial for controlling the level of AngII. Thus, due to the imbalance of ACE1 and ACE2 induced by virus infection, the hypertensive population may tend to experience more serious organ injury.

In hypertensive COVID-19 patients, more severe clinical types or mortality were observed, suggesting that hypertension might be associated with the clinical outcomes of COVID-19. Our study also found that 31.0% of hypertensive patients also had other forms of cardiovascular disease, which has been associated with an increased risk of death in COVID-19 patients^13,20^. However, hypertension remains the most important risk factor leading to cardiovascular disease^21^. Accordingly, preexisting hypertension, rather than cardiovascular disease, might be the underlying cause of increased susceptibility to rapid disease progression and more severe COVID-19.

Laboratory results provided some evidence for this hypothesis. Hypertensive COVID-19 patients presented elevated levels of serum urea nitrogen, serum creatinine, lactate dehydrogenase, creatine kinase, and NT-proBNP and markedly reduced eGFR at admission. These altered parameters indicate that internal organs with high levels of ACE2 protein expression, such as the lung, kidney, and heart^3,22^, are more vulnerable to invasion and injury by SARS-COV-2. Simultaneously, SAA and CRP, which reflect systemic inflammation throughout the body, were also elevated. These results indicate that hypertensive patients tended to develop more severe COVID-19 not only through serious cytokine storms but also through reduced protection against organ injury due to imbalances in the ACE system^23–25^. Thus, the delay from illness onset to hospital admission could result in an increased risk of severe illness or death in hypertensive patients with COVID-19.

More dynamic changes were observed in hypertensive COVID-19 patients. White blood cell and neutrophil counts remained higher in the hypertension group than in the nonhypertension group for almost the entire period of hospitalization; SAA and CRP continued to increase until approximately 10 days after admission. In contrast with the nonhypertension group, in the hypertension group, lymphocyte counts continued to decline until 10 days after admission and returned to the normal range 2 days later. These findings suggested that more serious cytokine storms occurred in hypertensive COVID-19 patients.

Notably, in addition to ACE2, other receptors on the surface of human cells can facilitate the entry of SARS-COV-2, including TMPRSS2^26^, sialic acid receptors^27^, and CD147^28^. Intriguingly, all of these factors are expressed by endothelial cells. Therefore, the endothelium, as one of the largest organs in the human body, is a key target organ in COVID-19^29^. Impairment of endothelium function can promote vasodilation, fibrinolysis, and antiaggregation^30–32^. Simultaneously, for hypertensive populations, endothelial dysfunction is also a key determinant of hypertension development and progression^33,34^. Therefore, hypertensive patients infected by SARS-COV-2 are vulnerable to more serious endothelial dysfunction and thus tend to develop a more severe COVID-19 phenotype, leading to a higher mortality rate.

In this study, 76.8% of patients were given antiviral agents based on previous experience in treating other coronavirus infections, such as SARS and MERS. Anti-influenza drugs, including oseltamivir and arbidol, were also used for empirical treatment during this unusual period. However, there are currently no agents proven to be an effective therapy for COVID-19^35,36^. Glucocorticoid therapy was also empirically used for severe complications in nearly 90% of patients. Nevertheless, the efficacy of glucocorticoids remains controversial because of their adverse effects^37^ and lack of consistently positive outcomes^4,38^. Recently, evidence from a randomized controlled trial showed that treatment with dexamethasone resulted in lowered mortality in COVID-19 patients^39^. Therefore, more reasonable glucocorticoid administration is still needed for COVID-19 treatment. Antibacterial therapy was also used in over 50% of patients to prevent or treat bacterial infections. Combined with the dynamic laboratory outcomes, parameters related to inflammation or organ function tended to improve 8–10 days after admission. However, these improvements could not be attributed to medication treatments alone.

This study had several limitations. First, only 82 patients with confirmed COVID-19 were included in the full analysis. A larger study population size would be helpful to more deeply understand the role of hypertension in the progression of COVID-19. Second, due to the retrospective study design, some information was unavailable, particularly the time from illness onset to the signs and symptoms in electronic medical records. Third, the positive rate of SARS-CoV-2 RNA detection in throat swabs is lower than that of other methods, and antibody assays were not available to assist in diagnosis by the end of January, resulting in the exclusion of suspected but unconfirmed cases from our analysis. Moreover, there are other potential risk factors, including age, chronic lung disease, cardiovascular disease, and even liver disease, that could lead to more severe disease and increased in-hospital death. However, this study initially identified hypertension as an important factor for the clinical outcomes of COVID-19. Further investigations of the mechanism in hypertensive populations are needed.
